# Task-Dependent Intermuscular Motor Unit Synchronization between Medial and Lateral Vastii Muscles during Dynamic and Isometric Squats

**DOI:** 10.1371/journal.pone.0142048

**Published:** 2015-11-03

**Authors:** Maurice Mohr, Marius Nann, Vinzenz von Tscharner, Bjoern Eskofier, Benno Maurus Nigg

**Affiliations:** 1 Human Performance Laboratory, Faculty of Kinesiology, University of Calgary, Calgary, Alberta, Canada; 2 Biomechanigg Sport & Health Research Inc., Calgary, Alberta, Canada; 3 Digital Sports Group, Friedrich-Alexander University Erlangen-Nuernberg, Erlangen, Germany; Universite de Nantes, FRANCE

## Abstract

**Purpose:**

Motor unit activity is coordinated between many synergistic muscle pairs but the functional role of this coordination for the motor output is unclear. The purpose of this study was to investigate the short-term modality of coordinated motor unit activity–the synchronized discharge of individual motor units across muscles within time intervals of 5ms–for the Vastus Medialis (VM) and Lateralis (VL). Furthermore, we studied the task-dependency of intermuscular motor unit synchronization between VM and VL during static and dynamic squatting tasks to provide insight into its functional role.

**Methods:**

Sixteen healthy male and female participants completed four tasks: Bipedal squats, single-leg squats, an isometric squat, and single-leg balance. Monopolar surface electromyography (EMG) was used to record motor unit activity of VM and VL. For each task, intermuscular motor unit synchronization was determined using a coherence analysis between the raw EMG signals of VM and VL and compared to a reference coherence calculated from two desynchronized EMG signals. The time shift between VM and VL EMG signals was estimated according to the slope of the coherence phase angle spectrum.

**Results:**

For all tasks, except for singe-leg balance, coherence between 15–80Hz significantly exceeded the reference. The corresponding time shift between VM and VL was estimated as 4ms. Coherence between 30–60Hz was highest for the bipedal squat, followed by the single-leg squat and the isometric squat.

**Conclusion:**

There is substantial short-term motor unit synchronization between VM and VL. Intermuscular motor unit synchronization is enhanced for contractions during dynamic activities, possibly to facilitate a more accurate control of the joint torque, and reduced during single-leg tasks that require balance control and thus, a more independent muscle function. It is proposed that the central nervous system scales the degree of intermuscular motor unit synchronization according to the requirements of the movement task at hand.

## Introduction

Human movements, particularly those of elite athletes and musicians, reveal substantial precision and coordination of the neuromuscular system. Motor unit (MU) synchronization is one feature of neuromuscular coordination that has been observed within a muscle, but also between synergistic muscles that are functionally and anatomically related [1–3]. Intermuscular MU synchronization (IMUS), the synchronized discharge of individual MUs between two muscles, is thought to originate from a common input of the central nervous system to branched presynaptic fibres that innervate motor unit pools across two muscles [2,3].

Despite frequent evidence for the presence of IMUS, its function with respect to task performance and force output is not well understood [4]. The existence of a functional role of IMUS is apparent from previous studies showing differences in synchronization between skill- and strength-trained individuals [5], increase in synchronization with increasing age [6] as well as task-dependency of IMUS [7]. The functional role of IMUS may remain unclear because the current knowledge is mostly based on evidence from isometric muscle contractions that are uncommon and not characteristic of typical human movement [8]. In our view, IMUS plays a minor role during isometric contractions but becomes more beneficial during movement, i.e. during tasks that involve changes in the length of multiple synergistic muscles and thus require greater synchronous control.

Accordingly, MU synchronization between individual muscle compartments of the medial gastrocnemius is higher during dynamic compared to isometric contractions [9,10]. In this previous study, MU synchronization was investigated using a coherence analysis between raw, monopolar surface electromyograms, obtained by a newly developed current amplifier [11]. The advantage of measuring EMG signals using a current rather than a potential amplifier is that there should be no lateral currents between adjacent electrodes limiting the risk of inter-electrode cross-talk.

Based on the finding of the previous study that intramuscular MU synchronization is task-dependent [9], we hypothesized that a task-dependent MU synchronization can also be revealed between two individual synergistic muscles sharing anatomical and functional features. Specifically, if two muscles act in concert to control a joint, such as Vastus Medialis (VM) and Vastus Lateralis (VL), we speculate that MU activity must be highly coordinated. The result that about 40% of VM and VL MUs already synchronize during isometric knee extensions substantiates this assumption [12].

The purpose of this study was to investigate (1^st^) whether IMUS between the VM and VL can be revealed using a coherence analysis between raw monopolar EMG currents and (2^nd^) whether the strength of IMUS is task-dependent. We hypothesized (H1) that there would be significant coherence between monopolar EMG currents of VM and VL representing MU synchronization and (H2) that IMUS would be highest during dynamic contractions of the VM and VL and lowest during isometric and balancing tasks. Balancing tasks may require a more independent MU activity.

## Methods

### Participants

Sixteen healthy, male (n = 12) and female (n = 4) participants (mean±SD; age 26±5 y) volunteered and gave their written informed consent to participate in this study. Ethical approval for this research study involving human participants was obtained from the University of Calgary’s Conjoint Health Research Ethics Board, in spirit of the Helsinki Declaration.

### Experimental procedure

**Fig 1:**
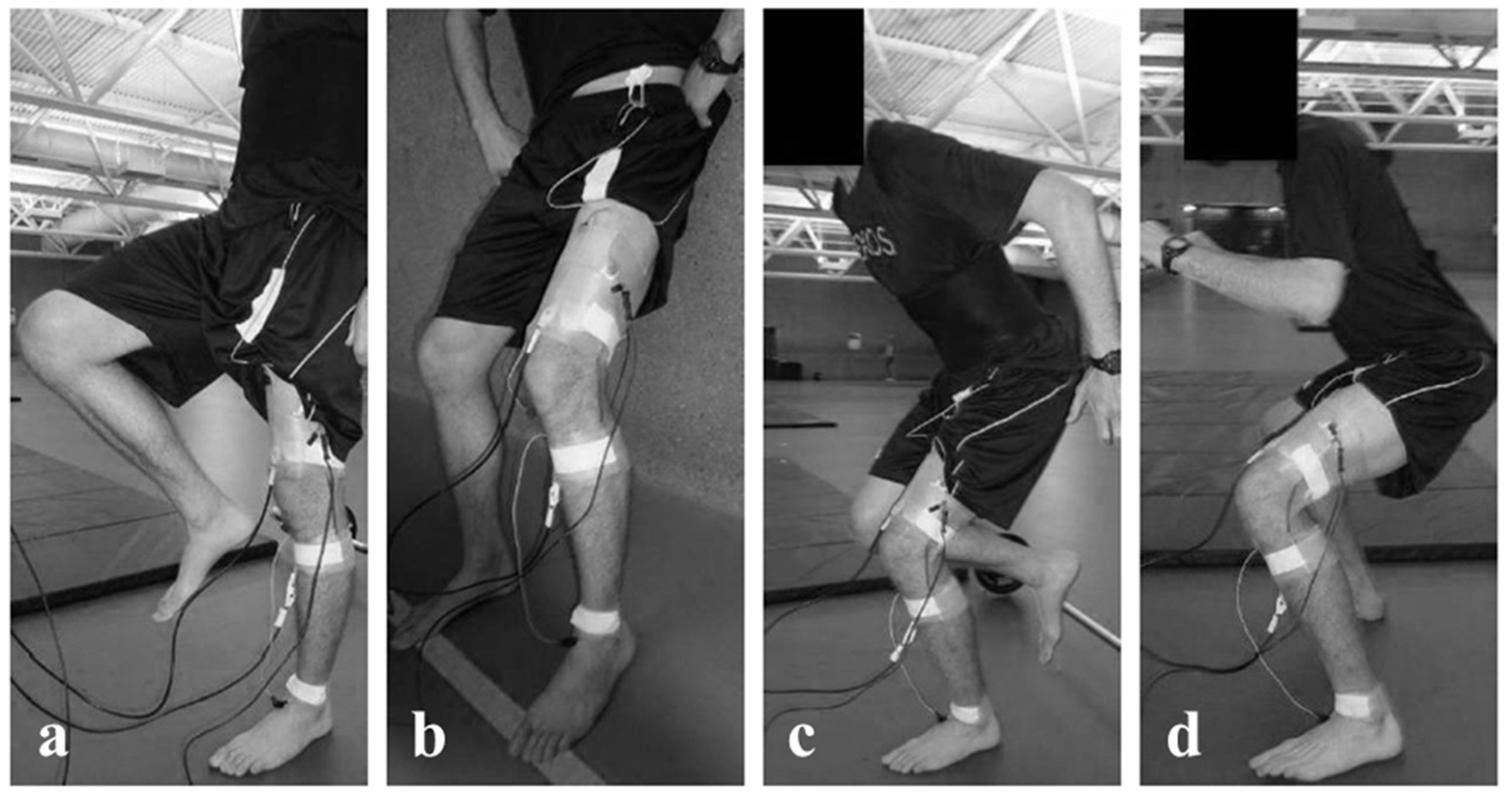
Movement tasks. Single-leg balance (SLB) (a), isometric squat (ISO) (b), single-leg squat (SLS) (c), bipedal squat (BPS) (d).

Each participant performed four different tasks each lasting 60 seconds: Bipedal squats (BPS), single-leg squats (SLS), an isometric squat (ISO), and single-leg balance (SLB). For the BPS, participants performed a series of squats down to a knee flexion angle of 70 degrees with their feet more than shoulder wide apart (Fig 1D). For the SLS, participants accomplished a series of squats down to a knee flexion angle of 45 degrees while balancing on the left leg (Fig 1C). For both, BPS and SLS, the squatting speed was set to 20 squats per minute and controlled for by using a metronome. In order to ensure consistent knee flexion angles, participants were given visual real-time feedback from a one-dimensional electrogoniometer (Biometrics Ltd., UK) taped across the lateral aspect of the participants’ knee joint. For the ISO, participants leaned with their back against a wall and held their lower legs perpendicular to the ground with their knees flexed at an angle of 45 degrees (Fig 1B). For the SLB, participants were asked to balance on their left leg while trying to keep an upright upper body posture (Fig 1A).

### EMG signal recording

**Fig 2:**
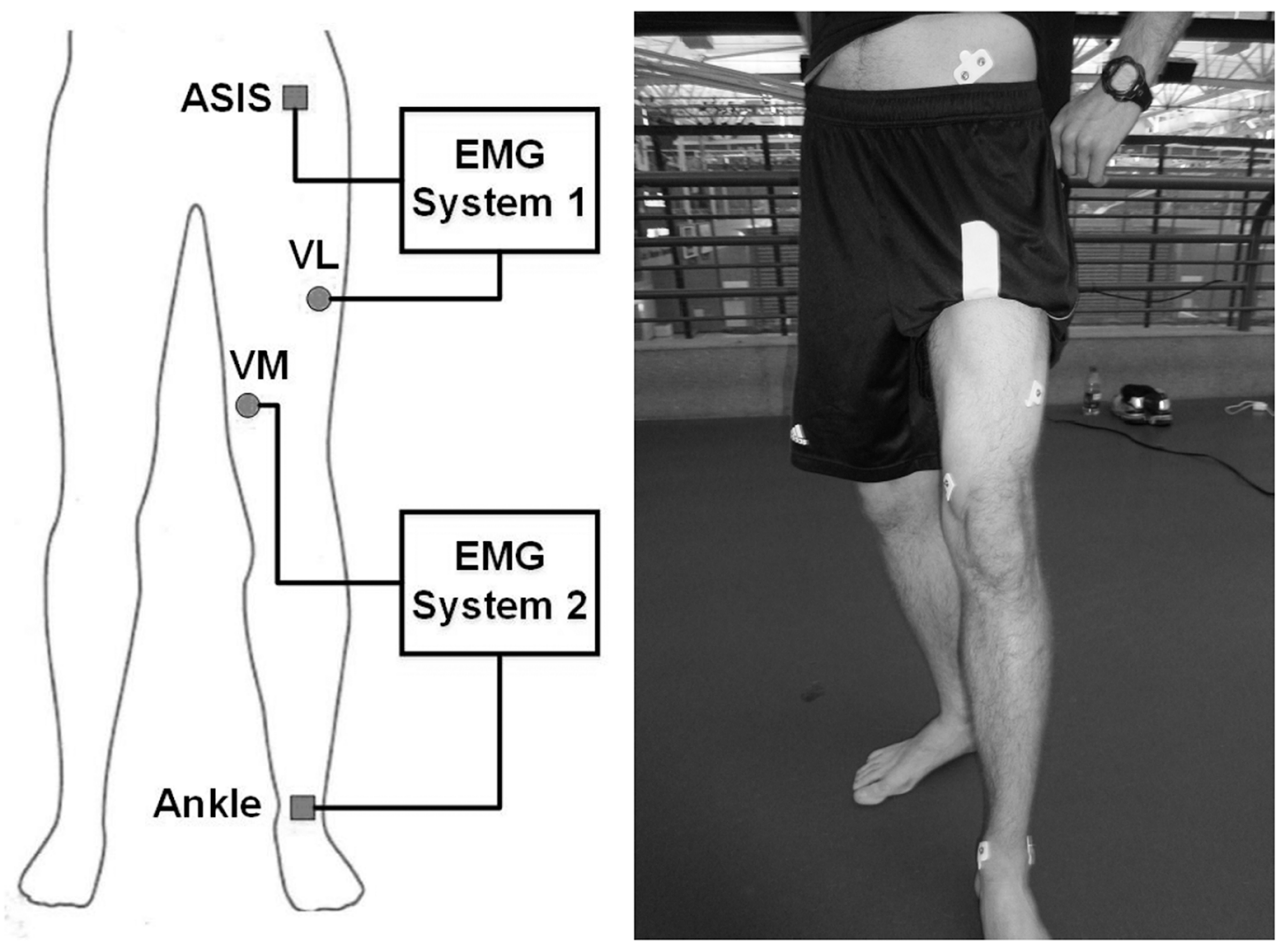
EMG recording set-up.

In order to obtain monopolar EMG currents from VM and VL, the skin surface above the muscles was shaved, slightly abraded with sand paper and cleaned with alcohol wipes to ensure high signal conductivity. Monopolar Ag-AgCl electrodes (Norotrode Myotronics-Noromed Inc., US) were placed over the muscle bellies of VM and VL according to electrode locations recommended in SENIAM guidelines [13]. One has to be aware that if commonly used bipolar electrodes are placed on non-fusiform muscles, such as VM and VL, each electrode records the activity of several motor units. Since MUAPs of the same muscle are often synchronized [1,10], both electrodes may simultaneously record similar signals. In a bipolar set-up these similar signals would be fully or partially eliminated by the common-mode rejection of differential amplifiers [9]. Therefore, the monopolar electrode set-up was chosen to obtain the EMG signal in its full complexity and extent. EMG currents were recorded using modified models of a previously described monopolar current amplifier [11]. Comparisons between EMG signals recorded with a current and a traditional potential amplifier demonstrated the validity of current recordings [11]. Modifications to the amplifiers included capacitive coupling (5μF) between the measuring electrode and the amplifier to filter out DC offset currents as well as a reduction of the trans-impedance resistance from 2.2MOhm to 50kOhm. The trans-impedance resistance (R) sets the amplification factor of the amplifier (U = R•I). Both of these modifications were made to avoid saturation of the amplifiers during recording. The signals were then electronically band-pass filtered between 10 and 500Hz. In order to avoid cross-talk between the two amplifiers, EMG recordings of each muscle were obtained using two separate recording systems with separate ground electrodes, data acquisition cards and battery powered laptops. Thus, the systems consisted of two electronically separated circuits. In system 1, EMG currents of VL were recorded using one measuring electrode above the VL and fed back over two ground electrodes placed side by side on the anterior superior iliac spine. In system 2, the EMG currents of VM were recorded using one monopolar measuring electrode above the VM and fed back over two ground electrodes placed on the medial and lateral malleoli (Fig 2). Two ground electrodes were used in each system to further reduce the resistivity to the returning currents. The two systems were synchronized using a custom built device that simultaneously transmitted a pulse to both systems upon pressing a button at the beginning and end of each measurement. EMG currents were sampled at a frequency of 2400Hz, thus avoiding aliasing.

### Signal processing

**Fig 3:**
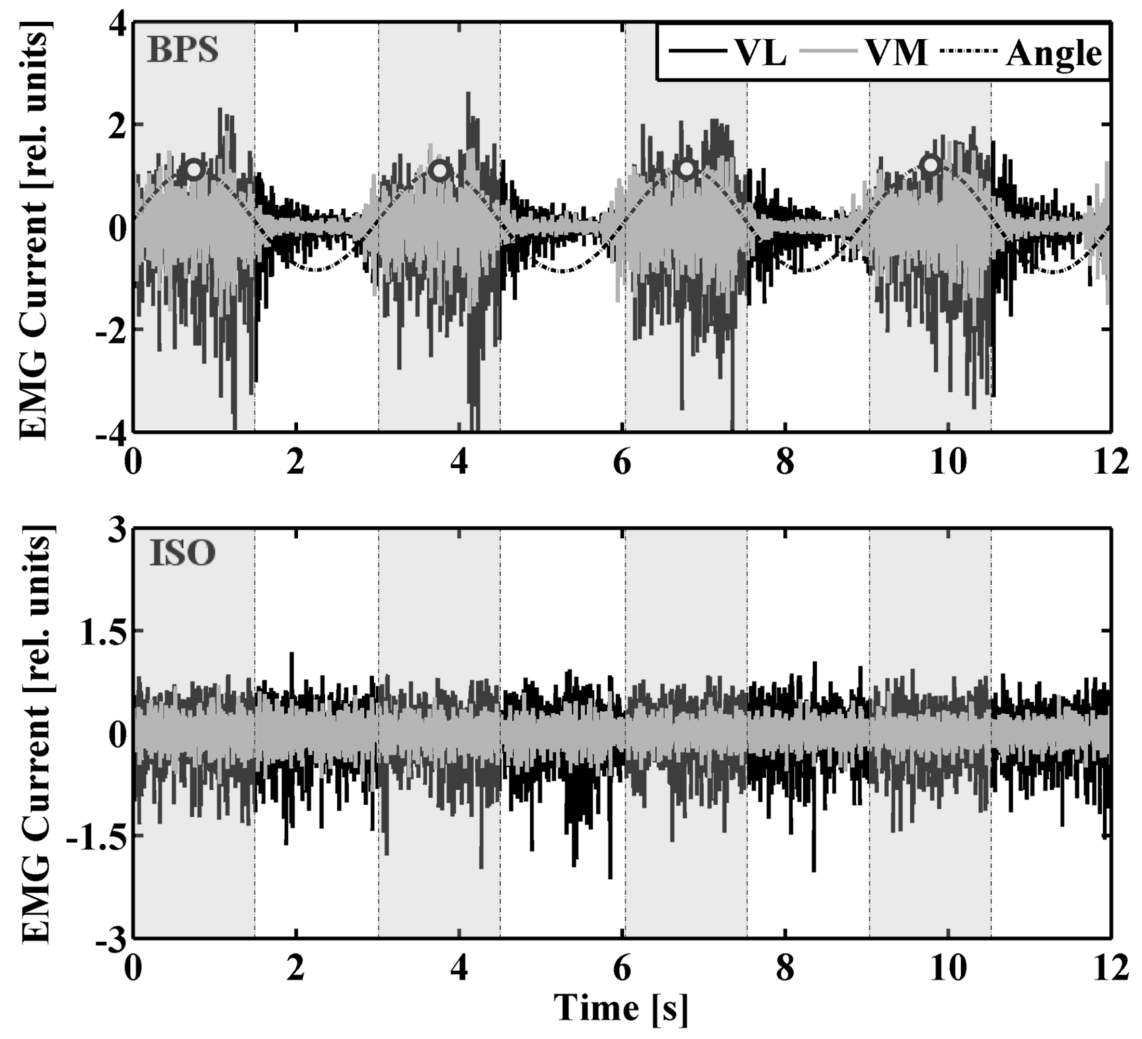
Procedure to separate individual data sequences according to peaks in knee flexion angle. Example for a bipedal squat (top panel) and corresponding isometric squat (bottom panel). Dashed vertical lines to the left and right of peaks in the knee flexion angle trace indicate the boundaries of individual data sequences (shaded) that were used for further analysis.

Goniometer data were low-pass filtered (cut-off frequency of 0.3Hz) using a wavelet-based filter method. The 60Hz line-frequency contamination was removed from the EMG currents by applying a line-frequency averaging method and a line filter. In short, this procedure allows to subtract the average line-frequency contamination from the EMG signal without inducing a notch in the EMG power spectrum at 60Hz (see [11] for further details). Removing the line-frequency from the signals avoided an artificial MU synchronization at 60Hz. For BPS and SLS, the signals were separated into sequences of 3584 samples (1.5 s) according to peaks in the goniometer signal that represented the time points of highest knee flexion during the squats. While these sequences contained the majority of the EMG power during the squats (Fig 3), the exact sequence size facilitated using a Fast Fourier Transform (FFT) during the analysis. EMG current signals recorded during ISO and SLB were separated into equally sized sequences using the same intervals as for BPS and SLS, respectively (Fig 3). After removing data of the first and last squat, 17 sequences of EMG currents from VM and VL were retained for further analysis for each task and participant.

### EMG signal analysis

The coherence analysis was applied to the unrectified EMG currents recorded for each sequence of squatting or isometric task. The data sequences were segmented into 7 non-overlapping windows with a length of 2^9^ = 512 time points, leading to a frequency resolution of 4.7Hz. The FFT was computed for each window *n*, yielding the Fourier spectra of $F_{{VL}_{n}}(\lambda )$ and $F_{{VM}_{n}}(\lambda )$. For each sequence *s*, i.e. one individual squat or isometric sequence, the coherence and the phase angle *φ* as a function of frequency *λ* (coherence and phase angle spectrum), were computed from the average cross-spectra normalized by the corresponding auto-spectra across all windows [14]:

$$coherence_{s}(\lambda )=\frac{{\left|\bar{F_{VL_{n}}(\lambda )\cdot F_{VM_{n}}{\left(\lambda \right)}^{*}}\right|}^{2}}{\bar{\left(F_{VL_{n}}(\lambda )\cdot F_{VM_{n}}{\left(\lambda \right)}^{*}\right)}\cdot \bar{\left(F_{VL_{n}}(\lambda )\cdot F_{VM_{n}}{\left(\lambda \right)}^{*}\right)}}$$ (1)

$$\varphi_{s}(\lambda )={\text{tan}}^{-1}(\bar{\left(F_{{VL}_{n}}\left(\lambda \right)\cdot F_{{VM}_{n}}{\left(\lambda \right)}^{*}\right)})$$ (2)

In the presence of a temporal delay between the EMG currents of VM and VL, the phase angle increases or decreases linearly across frequencies. The temporal delay is equal to the phase angle slope multiplied by 1/2π, where a negative gradient indicates a leading reference signal [15]. For this analysis, the VL was selected as the reference signal. For each task and participant, the average coherence and phase angle spectra between 15–100Hz were computed across all individual sequences, i.e. across 17 squats or 17 isometric sequences. The frequency range was selected based on the observation that 95% of the power of VM and VL was contained between 15–100Hz. The coherence of interest (*CoI*) for each participant and task was calculated as the sum over the average coherence spectrum between 30–60Hz and reported as a percentage of perfect synchronization, i.e. a constant coherence of 1. The frequency range of 30–60Hz was chosen since coherence in this range was highest during each of the movement tasks. The frequency at the peak of the coherence spectrum (*FPC*) was determined for each task and participant.

**Fig 4:**
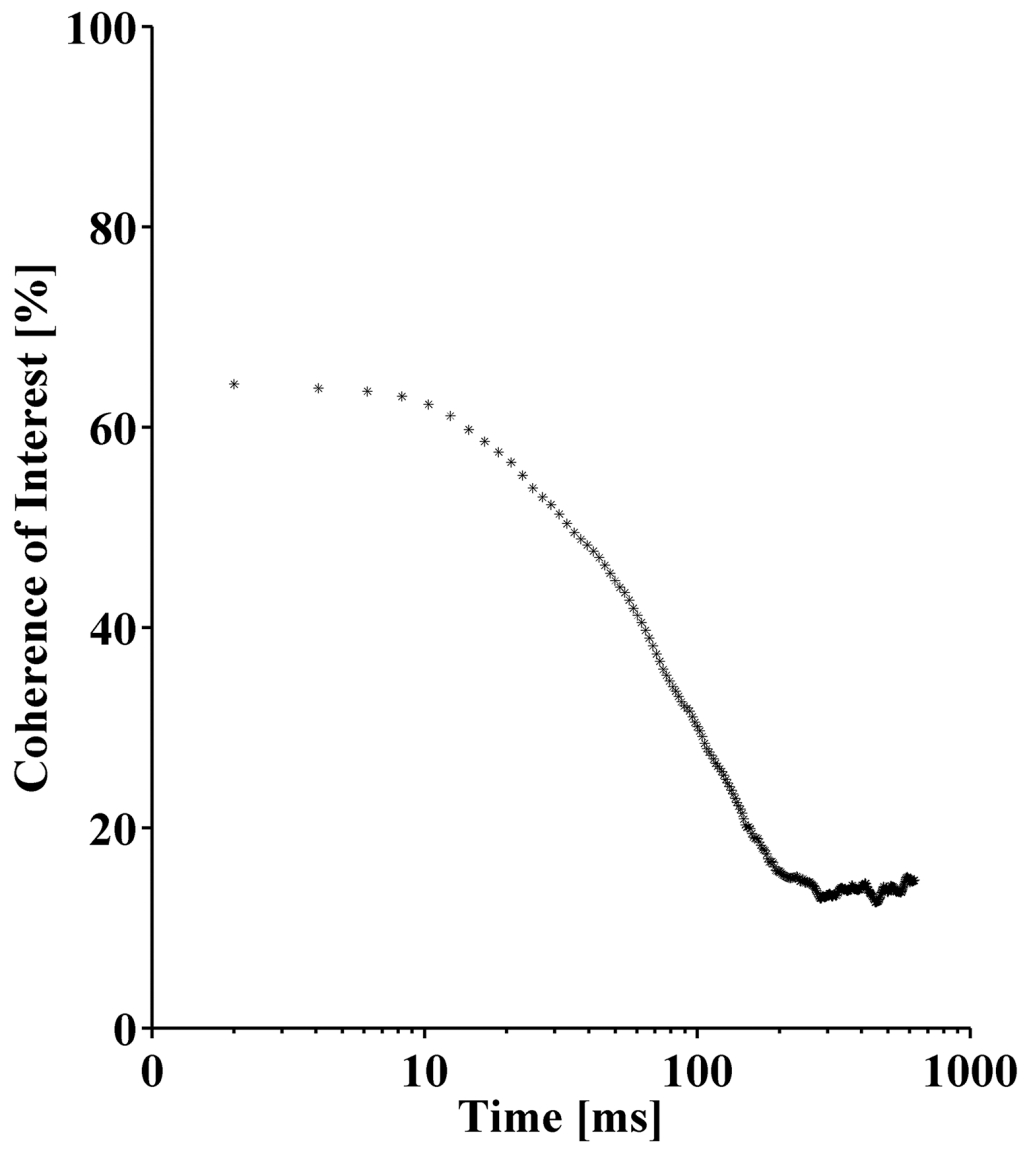
Decay of coherence of interest (*CoI*) with increasing time shift during a bipedal squat.

In order to evaluate the significance of the computed coherence spectra, a reference coherence was established. While the coherence analysis still yields high values for small time shifts between two signals, the coherence approaches values close to zero for time shifts exceeding some cycle durations. In an iterative approach, the EMG signal of the VM was shifted by j ms with respect to the VL signal with j increasing from 2 to 625ms. For each individual time shift, *CoI* was computed in the same way as described above. The coherence decay with increasing time shift is displayed in Fig 4 and was used to determine the time shift, at which two EMG signals can no longer be considered coherent. Fig 4 demonstrates that the *CoI* reached a constant minimum for time shifts exceeding 200ms. A greater time shift of 400ms was chosen for this study to ensure that the reference coherence was indeed at its minimum.

The surface electromyogram is known to be a superposition of individual motor unit action potentials (MUAPs). When MUAPs occur at almost the same time within a time window of 5ms, i.e. short-term MU synchronization [1], the coherence between two EMG signals will be elevated at frequencies common to the MUAP power spectrum. In addition to short-term synchronization of individual MUs, previous research has frequently shown coordinated rhythmic oscillations in the global muscle activation intensity [16–18]. Such a rhythm can only occur when MUAPs cluster at the frequency of the oscillation. However, this MU clustering is a different aspect of neuromuscular control that may not necessarily require short-term MU synchronization with accuracy of 5ms and may occur at different frequencies. Therefore, MU synchronization and MU clustering have to be separated and understood as two different phenomena. In order to emphasize the difference between IMUS and coordinated oscillations of the global muscle activation intensity between VM and VL, an additional analysis was conducted for one representative participant during the BPS task. To this purpose, the signal power of VM and VL was resolved in time and frequency space using a wavelet approach. The wavelet analysis was performed using 13 non-linearly scaled wavelets with centre frequencies ranging from 6.9Hz to 542Hz [19]. Wavelet data was separated according to the individual squatting sequences and converted into an intensity pattern, displaying the intensity (square root of power) of muscle activation of VM and VL in time and frequency simultaneously [20]. The total intensity, representing the level of global muscle activation, was calculated as the sum over wavelets 2–5, corresponding to centre frequencies of 19Hz to 92Hz, respectively. The total intensity is a close approximation of the commonly used EMG root mean square (RMS) [19]. The total intensities of VM and VL were subjected to the same coherence analysis as described above, thus revealing the coherence of oscillations of the global muscle activity.

In addition to coherence analyses, the global muscle activation intensity was compared across movement tasks to estimate Vastii muscle force demands and to discuss potential effects of varying muscle force demands between tasks on the degree of IMUS. The total intensities of VM and VL were summed across each sequence to compute the overall EMG intensities during a squatting or isometric task. The overall EMG intensity was averaged across sequences, and expressed as a percentage of the overall EMG intensity during the SLS.

### Statistical analysis

For each task, the mean and standard error of the average coherence and phase angle spectra between VM and VL were computed across all participants. In addition the mean and standard error of *CoI*, *FPC*, and the relative overall EMG intensities of VM and VL for each task were calculated. Each discrete variable was evaluated regarding normality using a Shapiro-Wilk test and Q-Q normality plots. A repeated measures ANOVA with the within-subject factor ‘task’ was performed to detect significant differences in *CoI*. Mauchly’s test of sphericity was used to test the assumption of sphericity. If the assumption of sphericity was violated, the Greenhouse-Geisser correction was used. Bonferroni corrected post-hoc tests were carried out to determine pairwise comparisons between individual tasks. Due to a deviation from normality, task-dependency of *FPC* was tested using a Friedman two-way ANOVA and medians are reported. All statistical tests were carried out using IBM SPSS statistics (v.20; SPSS Inc., Chicago, IL).

## Results

### Coherence and phase angle

**Fig 5:**
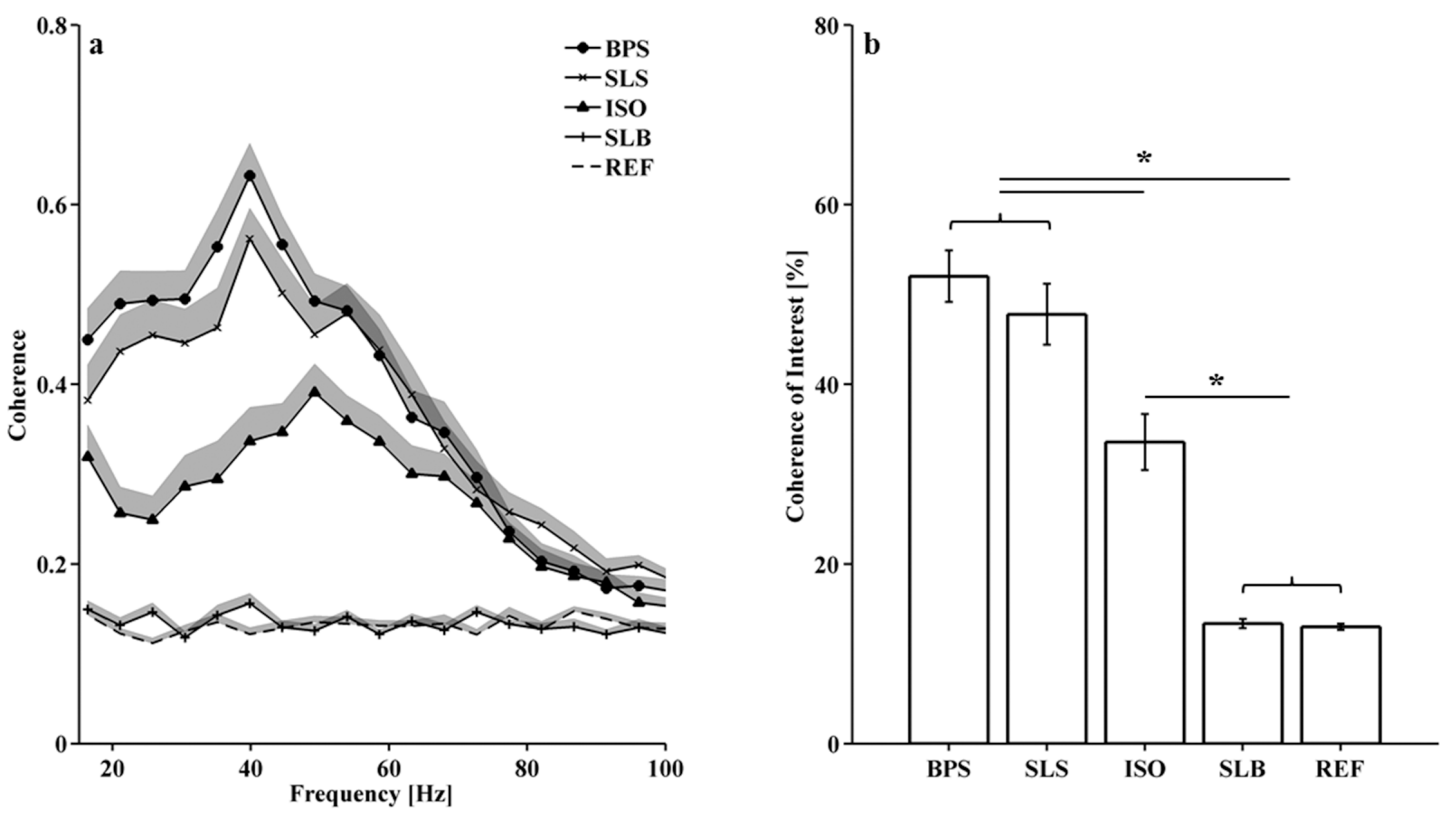
Task-dependent coherence between VM and VL EMG currents. Mean coherence spectra (+SE) (a) and coherence of interest (±SE) (b) across 16 participants for each task; asterisks indicate significant differences at α = 0.05.

The mean and standard error of the coherence spectra during each task are displayed in Fig 5A and 5B, respectively. The reference coherence was constant across frequencies with values between 0.1 and 0.2. Between 15–80Hz, the coherence spectra for each task showed higher values compared to the reference coherence, except for SLB (Fig 5A). For SLB, a difference to the reference coherence was undetectable. The coherence during BPS, SLS, and ISO was highest between 40Hz and 60Hz, with peak coherence occurring at higher frequencies for the ISO. The highest coherence spectrum was found for the BPS, followed by SLS, ISO and SLB.

Accordingly, the ANOVA indicated a significant main effect of ‘task’ on *CoI*, F(2,27) = 104, p<0.001. Post-hoc comparison showed that *CoI* was significantly higher during BPS (mean±SE, 52±3%, p<0.001), SLS (48±3%, p<0.001), and ISO (34±3%, p<0.001) but not SLB (13±0.5% (p = 1.000) compared to the reference coherence (13±0.3%) (Fig 5B). In addition, *CoI* during BPS (p<0.001) and SLS (p<0.001) were significantly higher compared to *CoI* of ISO. The elevated *CoI* during BPS compared to SLS was not statistically significant (p = 0.564). The median *FPC* was 45Hz for BPS, SLS, and SLB and 54Hz for ISO. The majority, 13 out of 16 participants, showed a lower *FPC* for BPS than for ISO. However, differences in *FPC* across tasks were not statistically significant, χ2(3) = 5.224, p = 0.156.

**Fig 6:**
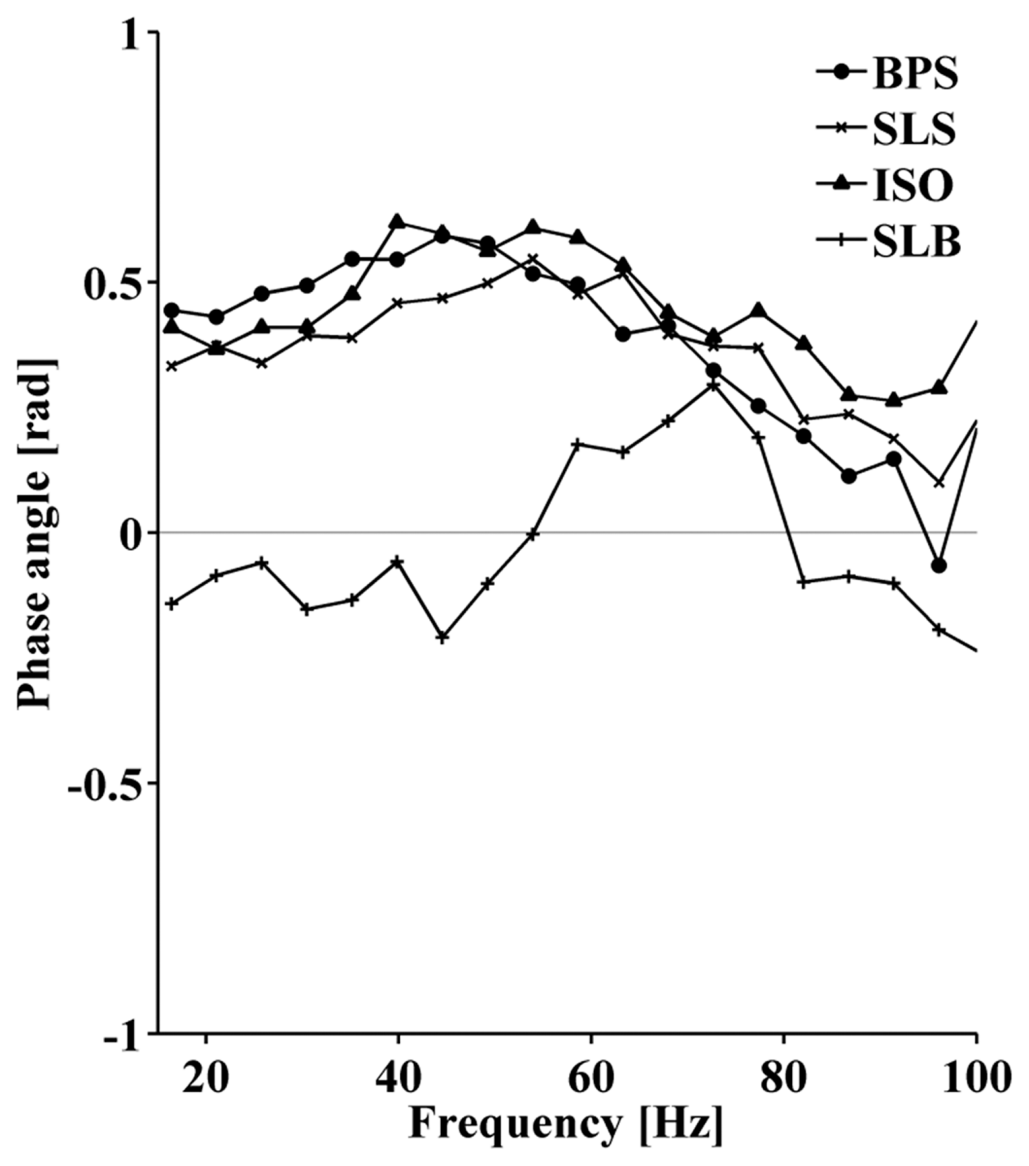
Phase angle between VM and VL EMG currents. Mean phase angle spectra across 16 participants for each task.

During BPS, SLS, and ISO, the phase angle increased from about 0.4rad to 0.5rad between 15Hz to 60Hz and then approached 0rad at frequencies above 80Hz (Fig 6). The slope of the phase angle in the frequency range used to compute *CoI* (30Hz to 60Hz) corresponds to an estimated time shift between VM and VL of 4ms, indicating a leading VM signal. The leading VM signal was consistent for 15 out of 16 participants. For the SLB condition, the phase angle remained close to zero.

### Coordination of global muscle activity

**Fig 7:**
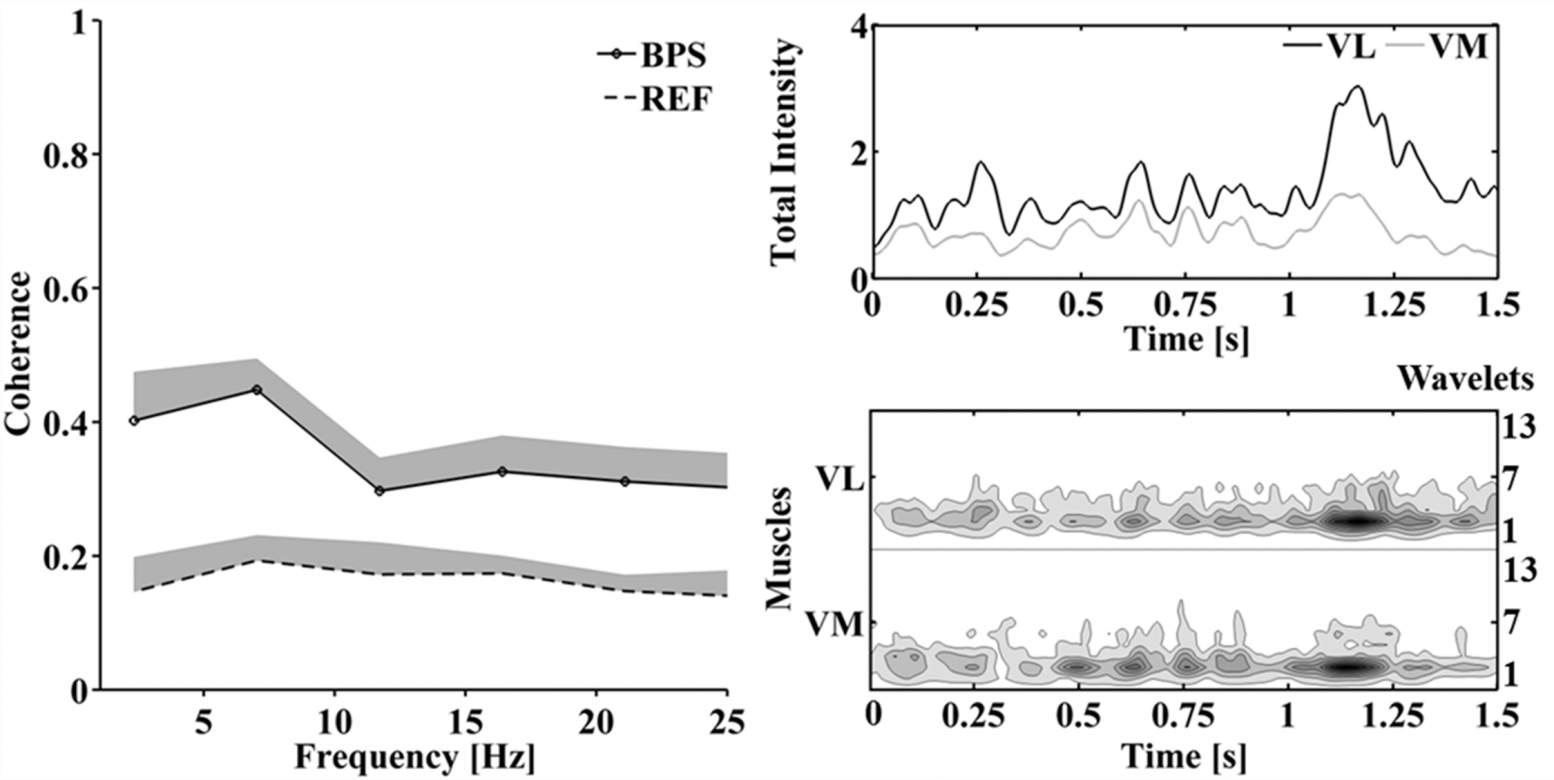
Coordinated oscillations in the global muscle activation intensity of VM and VL. Average coherence spectra (+SE) between total intensities of VM and VL during a bipedal squat (BPS) of one representative participant compared to the reference coherence (REF) (a); total intensities (b) and intensity patterns (c) of VL and VM during one bipedal squat.

The coordination of global muscle activity, as measured by the total intensity, is demonstrated for one representative participant. The VM and VL intensity was highest in wavelets 3 and 4, corresponding to the intensity of frequency bands centered at 38Hz and 62Hz, respectively (Fig 7C). From the total intensities of VM and VL it is apparent that the intensity bursts of VM and VL during the squat occurred at the same time (Fig 7B). The average coherence spectrum obtained for the total intensities of VM and VL across 17 BPS was higher than the coherence spectrum of a reference coherence from a time shifted condition. The coherence between the total intensities revealed a peak at 7Hz, thus at much lower frequencies than the *FCP* (Fig 7A).

### Overall EMG Intensity

**Fig 8:**
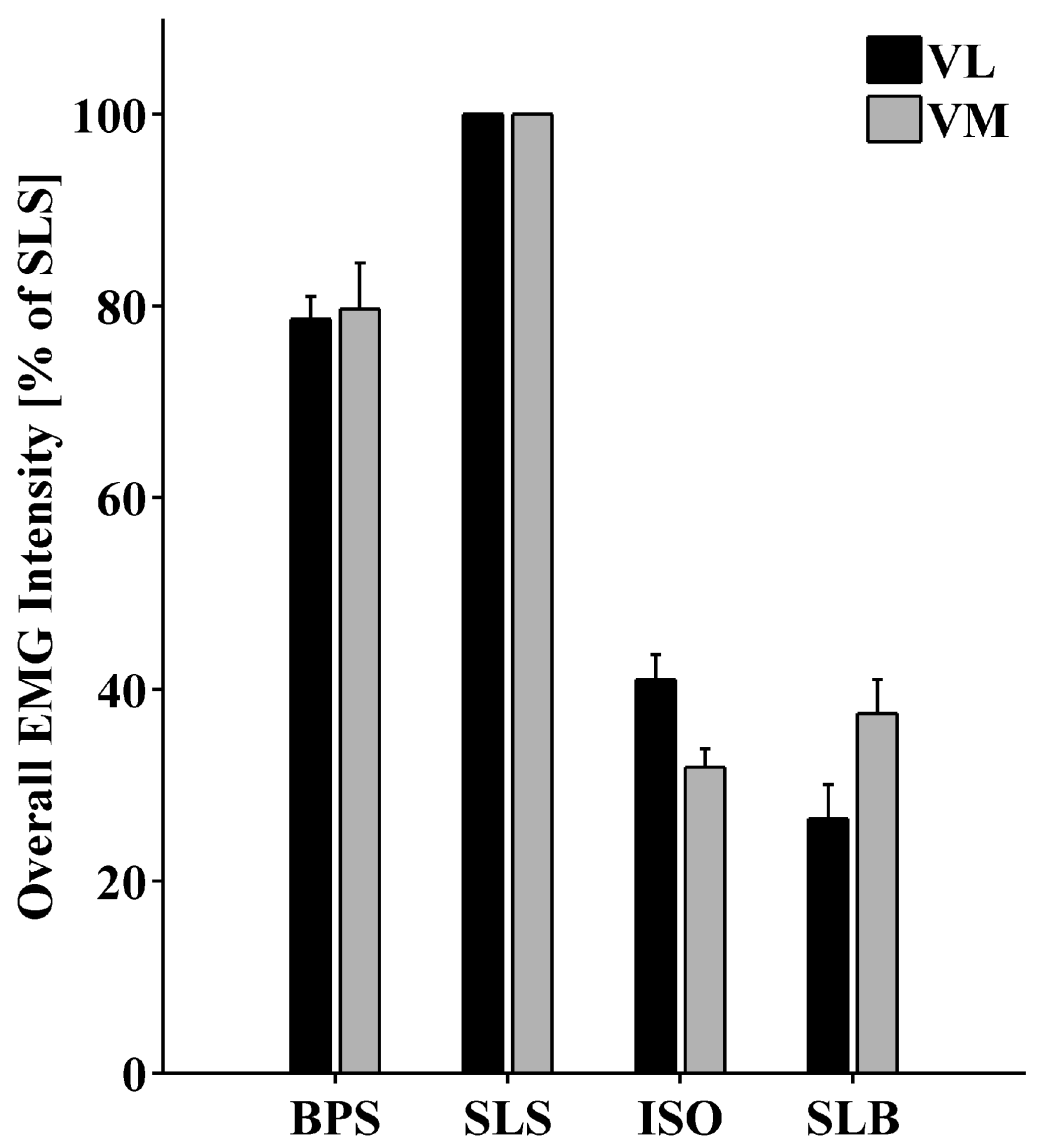
Overall EMG Intensity of VM and VL for each task. Graph shows mean values (+SE) normalized to the SLS across 16 participants.

The overall EMG intensity of VM and VL was maximal during the SLS for all participants (Fig 8). Relative to the SLS, the overall EMG intensity of both VM and VL was reduced to 80% during the BPS and further reduced to 30–40% during ISO and SLB.

## Discussion

This study investigated intermuscular motor unit synchronization (IMUS) between VM and VL and its task-dependency using a coherence analysis between unrectified EMG currents of these muscles. The results provide evidence in support of our first hypothesis (H1), that there would be significant coherence between monopolar EMG currents of VM and VL indicating MU synchronization. Furthermore, our second hypothesis (H2), that IMUS would be highest during dynamic contractions of the VM and VL and lowest during isometric and balancing tasks was confirmed.

In this study, IMUS between VM and VL was revealed by a coherence between their raw, monopolar EMG currents that significantly exceeds the reference coherence. The reference coherence was calculated from VM and VL EMG signals that were purposely desynchronized. During all movement tasks except single-leg balance, VM and VL EMG currents were coherent for frequencies between 15–80Hz. The coherence spectra approached the random reference for frequencies above 80Hz. This was likely due to the result that the main EMG power of VM and VL was contained at frequencies below 80Hz (wavelets 2, 3, and 4; Fig 7C). In addition, it is known that individual MUAPs have a characteristic fine-structure, represented by higher frequencies in the EMG power spectrum, that are most likely not correlated [10].

As apparent from Fig 4, a feature of coherence is that its value is constant for small time shifts of up to 10ms but decays with larger time shifts. The constant coherence for small time shifts is assumed to represent the short-term modality of MU synchronization [1]. However, low significant coherence between two EMG signals was still observed for time shifts in the range of 20 to 100ms. These time shifts clearly exceed the width of MUAPs. Thus it seems that, in addition to the short-term modality of MU synchronization, coherence reflects a long-term correlation between the EMG signals that remains detectable after shifting the signals substantially. The coordinated oscillations of the global muscle activation intensity, revealed by a coherence between VM and VL total intensities at low frequencies (Fig 7), are most likely the reason for observing the long-term modality. These two modalities may reflect the findings of De Luca et al. [1] who suggested that short-term synchronization of MUs occurs within a time window of 5ms while there is a long-term modality between 8 and 76ms. Since the present study allowed estimating a time shift of 4ms between VM and VL, the coherence between VM and VL EMG currents during squatting is assumed to predominantly represent short-term IMUS.

The findings of this study are in agreement with previous studies demonstrating coordinated MU activity between VM and VL based on intramuscular and surface EMG recordings [12,16,17,21]. The result that the VM consistently fires prior to the VL in a healthy population supports earlier results [22] and, thus show the validity of the conducted analysis. In addition, these findings support the notion that functionally and anatomically related muscles show MU synchronization [3,23]. However, the comparison to the results of other studies is not trivial due to different recording and analysis techniques and corresponding effects on signal properties, coherence, and coherence interpretation [15,24]. Mellor & Hodges [12] used intra-muscular recordings and a cross-correlation analysis to demonstrate IMUS between VM and VL during isometric knee extensions. The presence of IMUS between VM and VL during the isometric squat in the current study supports these results. While the direct comparison to results of a coherence analysis is not yet feasible, it is known that the size of the peak in the cross-correlogram is significantly correlated with the magnitude of coherence [2,5]. To the best of our knowledge, the current study is the first to study IMUS between quadriceps muscles during dynamic contractions using a coherence analysis of raw surface EMG currents.

Further studies investigated coordinated oscillations in the global muscle activation intensity between individual quadriceps muscles using a coherence analysis between two surface EMG linear envelopes [17] or rectified surface EMGs [16]. In both studies, EMG signals from individual quadriceps muscles were coherent at frequencies between 6–20Hz. In the current study, the coordination of global muscle activation was visible in the intensity patterns of VM and VL from one representative participant, showing equally spaced synchronous bursts of VM and VL intensity during a bipedal squat. In agreement with previous studies, these synchronous bursts resulted in a peak of the coherence between VM and VL total intensities at 7Hz (Fig 7). Coordinated oscillations in the global muscle activation must be a result of clustered MU activation across two individual muscles. However, short-term MU synchronization within intervals of less than 5ms may not be necessary when clusters form. Consequently, we suggest that coherence between signals representing global muscle activation predominantly reflect the long-term modality of coordinated MU activity and should be viewed as a result of motor unit clustering, rather than short-term MU synchronization.

### Task-dependency of synchronization–Dynamic vs. Isometric

The second objective of this study was to investigate task-dependency of IMUS between VM and VL. Task-dependency was investigated by comparing the coherence between four different tasks: Bipedal squat (BPS), single-leg squat (SLS), isometric squat (ISO), and single-leg balance (SLB). These movements were selected to compare IMUS between dynamic, isometric, and balancing tasks that are frequently applied in sports, exercise, and clinical settings. In addition to differences in the nature of the movements, these tasks also differed in muscular force demands for the Vastii muscles. Since increased muscle force may elevate intermuscular coherence [17], potential effect modification with respect to IMUS was addressed by analyzing the overall EMG intensity of VM and VL during each task. Naturally, the SLS required the highest muscle activation as the body weight has to be lifted using only one leg. However, coherence during SLS was lower compared to the BPS task for all participants. In addition, ISO and SLB did not differ considerably in overall EMG intensity but the coherence was much lower during the SLB compared to ISO. While these observations do not prove the absence of an influence of the muscle force magnitude on IMUS, they indicate that the nature of the task–dynamic, isometric, or balancing–predominantly modified IMUS in this study.

Our hypothesis of stronger synchronization during dynamic compared to isometric contractions was supported by the significantly greater coherence during the BPS and SLS compared to the ISO. This observation is in agreement with experiments in hand and lower leg muscles where intramuscular synchronization was enhanced during dynamic contractions compared to isometric contractions [7,9]. In addition to a higher coherence, 80% of the participants demonstrated a shift of the frequency at peak coherence to lower frequencies during BPS (45Hz) compared to ISO (54Hz). Based on the recent results of von Tscharner [9] and earlier results [25,26], we assume that this is due to a shift to lower frequencies, i.e. from 54Hz to 45Hz, in the individual power spectra of VM and VL, indicating a greater MU synchronization within the individual muscles. Therefore, it can be speculated that the common drive to the motor unit pools of VM and VL was stronger during dynamic contractions resulting in higher MU synchronization within and between VM and VL and subsequently higher coherence at a lower frequency. The enhanced MU synchronization between muscles has been proposed to be a strategy of the central nervous system to reduce the degrees of freedom of the musculoskeletal system during complex tasks [27]. Furthermore, one could argue that by increasing the strength of IMUS between VM and VL, the central nervous system facilitates a more synchronous control of medial and lateral muscle forces acting on the knee joint. This may be particularly important to maintain a balanced force development during dynamic tasks that involve length changes of multiple synergistic muscles. Indirect evidence for this argument is apparent from patients with anterior knee pain. Anterior knee pain has been suggested to originate from inaccurate coordination of medial and lateral muscle forces controlling the joint [28]. Correspondingly, patients with anterior knee pain showed lower IMUS between VM and VL compared to healthy controls [29]. However, the link between muscle force or joint control, joint disease, and intermuscular MU synchronization requires further research [4].

### Task-dependency of synchronization–Balance

Our second hypothesis, that MU synchronization would be lower if balance elements are induced was based on the assumption that during SLB and SLS the two Vastii muscles have to work more independently to maintain balance. This hypothesis was partially supported by the finding that a majority, 11 out of 16 participants, showed a higher coherence during BPS compared to SLS. However, the mean difference between tasks did not reach statistical significance, likely due to high between-subject variability. In addition, the coherence between VM and VL for SLB was not detectably different from the reference coherence of shifted signals. The reduced or diminished coherence, representing reduced IMUS, might constitute an attempt of the central nervous system to enhance a more independent muscle function of the VM and VL during a balance task such as SLS and SLB compared to bipedal tasks. In support of this assumption, Semmler and colleagues [5,30] demonstrated that IMUS between finger muscles is lower in skilled musicians who rely on a highly independent muscle function compared to weight-lifters and controls. During SLB no significant coherence between VM and VL was found. This finding indicates that during SLB, motor units of VM and VL may not synchronize at all or only synchronize to an undetectable extent. An explanation for the absence of IMUS between VM and VL may be that the single-leg balance task is predominantly accomplished by participants through synergistic activation of the muscles around the ankle and hip joint as opposed to the quadriceps [31]. In support of this explanation, Gibbs et al. [23] observed IMUS during standing tasks between the bi-articular Gastrocnemii and Hamstrings muscles, controlling the ankle and hip joint. Consequently, muscle forces of the VM and VL may only be required intermittently to regain balance in the medio-lateral direction. These muscle forces should be highly independent and benefit from low synchronization. Future studies have to further investigate the diminishing effect of balance tasks on intermuscular synchronization, possibly by comparing eyes-open to eyes-closed tasks.

### Limitations and other concerns

The biggest concern and limitation is caused by the non-stationary property of the EMG signal. Depending on the analysis method selected, the short-term or long-term modality of MU synchronization is more or less noticeable and separable. It seems that short-term synchronization can be observed in the cross-correlogram of intramuscular recordings or in the coherence spectrum of raw surface EMGs rather than in the coherence spectrum of rectified EMGs or EMG linear envelopes. This methodological difficulty could not be completely circumvented in the present study. The reason for analyzing the raw EMG signal was to primarily keep the focus on the short-term modality. The importance for future work is to pay more attention to separating the two modalities, possibly by combining regularity measures of EMG signals [32] with coherence analyses.

Before deriving conclusions from a coherence analysis, the risk of cross-talk between the electrodes and common mode noise should be evaluated due to their misleading effects on coherence [15]. Cross-talk can lead to artificially large coherence values at certain frequencies. This was addressed by using two separate measuring systems and was carefully considered but not observed in the current study. The effect of the presence of common mode noise on coherence was tested by adding the same low-amplitude EMG signal with a randomized phase to both the VM and the VL signal and re-calculating coherence. An inflated coherence spectrum in the presence of common mode noise was not observed. In addition, if any other common mode noise or cross-talk was present and was dominating the coherence between VM and VL, one would not expect the coherence to differ between tasks or the coherence to be absent during SLB. Therefore, it was unlikely that cross-talk or common mode noise modified any of the present results.

A limitation of the protocol used for this study was the variable Vastii muscle force, length and speed of shortening and lengthening between the four motor tasks, which may affect IMUS. Differences in muscle force levels were addressed above and did not seem to modify our results significantly. The effect of knee flexion angle and respective Vastii muscle length on IMUS between VM and VL has been investigated by Mellor & Hodges [33]. Since synchronization was shown to be independent of the knee flexion angle, we assume that different squatting depths in this study did not influence coherence. Finally, the Vastii shortening and lengthening velocity was higher during the BPS compared to the SLS due to a lower squatting depth at the same squatting speed. While increased shortening speed has been suggested to require stronger MU synchronization [4], Hansen et al. [21] did not observe an effect of different walking speeds and consequently different muscle shortening velocities on MU synchronization. Therefore, we are confident that the conclusions of this study with respect to the task-dependency of IMUS between VM and VL are valid despite the biomechanical differences between the motor tasks. Nevertheless, the goal of future research should be to systematically examine the effects of muscle length and speed of shortening on IMUS at varying force levels.

A technical limitation of this study was the short duration of individual dynamic squats leading to the low frequency resolution of 4.7Hz. This technical limitation did not allow investigating differences in IMUS throughout the time course of a squat, which may be important since IMUS is assumed to differ between concentric and eccentric contractions [7]. Future studies will be aimed at incorporating a wavelet-based coherence analysis that may allow a) to examine the time course of MU synchronization during squatting and b) may be able to discriminate the short and long-term modalities of coherence [34].

## Summary and Conclusions

This study demonstrated that substantial short-term motor unit synchronization is present between VM and VL at frequencies between 15–80Hz and can be revealed using a coherence analysis between raw surface EMG currents. It was shown that intermuscular motor unit synchronization is not equivalent to the correlation observed between the global muscle activity e.g. coherence of EMG envelopes. Coordinated oscillations in global muscle activity between two muscles require that motor units are activated in clusters at the frequency of the oscillation. However, the present study shows that there is a much tighter synchronization between motor units of individual muscles within a time frame of 5ms. Furthermore, synchronization of motor units between VM and VL was task-dependent with a) enhanced synchronization during dynamic compared to isometric squats and b) reduced synchronization when balance elements were induced. We speculated that the central nervous system controls the degree of synchronization by modulating the strength of a common input to the motor unit pools of VM and VL according to the requirements of the movement task at hand. It seems logical that humans should be able to individually control motor units and to activate, cluster and synchronize them only if necessary and energy wise appropriate. However, the degree of correlation between two raw EMG signals, which at a first glance appear quite random, is surprising. Future research should attempt to separate the short and long-term modalities of synchronization and associate them with functional outcomes such as performance, injury, or certain diseases.

## Supporting Information

S1 File Coherence, phase angle and overall EMG intensity for each participant. (XLSX)
